# Reproductive trends in females with inflammatory joint disease

**DOI:** 10.1186/s12884-016-0919-7

**Published:** 2016-05-31

**Authors:** Marianne Wallenius, Kjell Å. Salvesen, Anne K. Daltveit, Johan F. Skomsvoll

**Affiliations:** Department of Rheumatology, National Advisory Unit on Pregnancy and Rheumatic Diseases, Trondheim University Hospital, Trondheim, Norway; Department of Neuroscience, NTNU, Norwegian University of Science and Technology, Trondheim, Norway; Department of Obstetrics and Gynecology, National Center for Fetal Medicine, Trondheim University Hospital, Trondheim, Norway; Department of Laboratory Medicine, Women’s and Child Health, NTNU, Norwegian University of Science and Technology, Trondheim, Norway; Department of Global Public Health and Primary Care, University of Bergen, Bergen, Norway; Medical Birth Registry of Norway, Norwegian Institute of Public Health, Bergen, Norway

**Keywords:** Secular trends, Arthritis, Birth weight, Birth registry

## Abstract

**Background:**

The study assessed birth trends per decade in offspring of females with inflammatory joint diseases (IJD) compared with women without IJD.

**Methods:**

This retrospective cohort study is based on data from the Medical Birth Registry of Norway from 1967 to 2009. We investigated singleton births in females with IJD (*n* = 7502) and compared with births from the general population (*n* = 2 437 110). Four periods were examined: 1967–79, 1980–89, 1990–99 and 2000–09. In the logistic regression analysis adjustments were made for maternal age at delivery and birth order. Odds ratios were obtained for the associations between IJD and birth outcome for each period.

**Results:**

Females with IJD had in average 65 deliveries / year (0.08 % of all births) in the 1970ies and 274 deliveries / year (0.5 % of all births) from 2000 to 2009. Adjusted Odds ratios (aOR) for newborns small for gestational age were 1.5 (95 % CI 1.2, 1.9) in the earliest and 1.1 (95 % CI 0.9, 1.2) in the last period. Correspondingly, for birth weight < 2500 grams aOR decreased from 1.4 (95 % CI 1.0, 1.9) to 1.1 (95 % CI 0.9, 1.4). For preterm birth aOR was 1.1 (95 % CI 0.8, 1.5) in the first and 1.3 (95 % CI (1.1, 1.5) in the last period.

**Conclusion:**

An increasing number of births among females with IJD were observed in the study period. Birth weights of newborns of IJD women approached to birth weights in the general population, but preterm birth remained a problem.

**Electronic supplementary material:**

The online version of this article (doi:10.1186/s12884-016-0919-7) contains supplementary material, which is available to authorized users.

## Background

Inflammatory joint diseases (IJD), here including rheumatoid arthritis (RA), spondylarthritis (SA), psoriatic arthritis (PsA) and juvenile idiopathic arthritis (JIA) are characterised by chronic, systemic inflammation and may also affect internal organs. Previous studies have shown various effects of IJD on birth outcome [1–4]. Especially, low birthweight in offspring and preterm birth have been identified as prominent findings. Over time, improvements in diagnosis and treatment, and also change in registration of routines may influence birth outcomes [5, 6]. Before 1990 several females with IJD were dissuaded from having children [7, 8], and there were little experience with monitoring and medical treatment in pregnancy. In a study published in 1991 27 % of the women had been dissuaded from pregnancy by health professionals, family or other patients [7]. Since the end of the 1990ies new treatment options like the tumor necrosis factor (TNF)-α inhibitors have made more women able to achieve remission and thus enabled them to plan their pregnancies. Possible changes in pregnancy outcomes along with improved treatment options have not been published.

In the present study we wanted to assess secular trends in the number of births and the effects of IJD on preterm birth and birth weight in the newborns. The data were provided by the Medical Birth Registry of Norway from 1967 to 2009.

## Methods

### Material

The Medical Birth Registry of Norway (MBRN) is a population-based registry containing information on all births in Norway since 1967 comprising more than 2.4 million births 1967–2009. MBRN is based on compulsory notification of every birth or late abortion from 12 completed weeks of gestation onwards. Since 2002 MBRN has been organised under the Norwegian Institute of Public Health. A standard antenatal notification form is filled in at visits to a general practitioner, midwife or obstetrician during pregnancy and is brought by the mother to the place of birth. The midwife enters additional data recorded at the time of birth. Follow-up data are added to the form until discharge of hospital births. The attending midwife is responsible for the completion of the notification form at the end of each birth. The form is co-signed by the attending physician and sent to the MBRN within one week after birth. In the period January 1st 1967 to November 30th 1998 a notification form was used for all live births and stillbirths after 16 weeks of gestation. From December 1st 1998 a new and more comprehensive form was used for all live births and stillbirths after 12 weeks of gestation [9]. The new form intended to improve the registrations in MBRN and contains precoded fields for disease in mothers prior to pregnancy including rheumatoid arthritis. In addition there are open text fields for maternal diagnoses before and during pregnancy. The records include identification of the parents by their national identification numbers, demographic information of the parents, maternal diagnoses before and during pregnancy, parity, complications during pregnancy and delivery, length of pregnancy, as well as information on the infant, including birth weight, birth defects and other perinatal problems. The variables have been decided by consensus among obstetricians, neonatologists and epidemiologists. Information about parity is available for the index pregnancy, but also from any previous registrations in MBRN. In the present study we used the information from the index pregnancy, but if this was lacking, we used information based on previous registered pregnancies in MBRN.

Until December 1st 1998 diagnoses were recorded as stated by the International Classification of Diseases (ICD)-8 system. Afterwards the ICD-10 system has been used. It provides differentiation of the various diagnoses of IJD. Information on diagnoses was obtained from the patient’s doctor and from medical records. We have included data from females with IJD who gave birth in the period 1967 – 2009. The diagnoses of IJD were based on free text information in the birth registration form, as coded by MBRN using the ICD-8 or ICD-10 system, respectively.

The study was performed in compliance with the Helsinki Declaration and approved by the Regional Ethics Committee of Central Norway (diary number 2011/127-3).

### Exposure

We included anonymised cases with coding 712 in ICD-8, comprising rheumatoid arthritis (RA), juvenile idiopathic arthritis (JIA) psoriatic arthritis (PsA) and ankylosing spondylitis (AS). When the ICD-10 system was introduced we used M05.0, M05.1, M05.8, M05.9, M06.0, M06.8, M06.9 (RA), M45 (AS), M46.1 (spondylarthritis), L40.5 and M07.0, M07.1, M07.2 or M07.3 (PsA) and M08.0, M08.1, M08.2, M08.3, M08.4, M08.8, M08.9 (JIA). The different diagnoses within the IJD complex therefore had to be analysed as a collected group to obtain a comparison of the same diseases. We excluded unspecified arthritis, inflammatory connective tissue diseases and vasculitis in the selection of IJD patients (Additional file 1). All other singleton deliveries in females without IJD 1967–2009 served as reference group.

### Outcomes

Parity was defined as the total number of previous pregnancies registered per birth including spontaneous abortions after gestational week 16, live births and stillbirths. We also studied mean birth weight (BW) for the newborn, the proportion of newborns with BW < 2500 gram and the proportion born small for gestational age (SGA). SGA was defined as BW < 10 percentile for expected BW for gestational age. Gestational age at birth was based on ultrasound before 20 weeks or computed by the last menstrual period. Birth before 37 + 0 gestational weeks was defined as preterm.

### Statistical analysis

Births for females with IJD and reference deliveries were stratified into four periods: 1967–79, 1980–89, 1990–99 and 2000–09. Comparison of groups was studied by Student’s t-test for continuous variables and chi-square tests for categorical variables. Logistic regression analysis was used to assess associations between IJD and perinatal outcomes. We adjusted for parity and maternal age at delivery. Multivariable linear regression analysis was used to analyse mean differences in BW of the newborns of mothers with IJD and reference women. The explanatory variables were maternal age at delivery, gestational age, gender of infant and parity [10, 11]. Values of p that were <0.05 were considered significant. Data were analysed using the Statistical Package of Social Sciences, version 19.0 (SPSS Inc., Chicago, Illinois, USA) and Statistics / Data Analysis (STATA) version 12.1 (Stata Corp, Lakeway Drive College Station, Texas, USA).

## Results

From 1967 to 2009 a total of 7502 births were registered in females with IJD forming 0.3 % of totally 2.4 million births in Norway in the period. In the earliest period, 1967–1979, the births in females with IJD constituted 0.08 % of all births. This increased to 0.5 % in 2000–2009. Correspondingly, in average 65 deliveries annually were registered in females with IJD during 1967–1979, increasing to 274 each year after year 2000. In 2000–2009 (*n* = 2736 births) when detailed IJD diagnoses were available through ICD-10, the distribution of diagnoses was: RA 51 %, SA 32 %, PsA 10 % and JIA

7 %. The mean age at delivery for females with IJD was higher than among the reference women through all periods, and parity was higher from 1990 onwards. In the period 2000–2009 more females with IJD were smoking at time of conception than among the references (Table 1). For offspring of IJD mothers the crude mean birth weight (BW) was significantly lower than in controls through all periods. However, the mean BW difference decreased from −61 g in the 1970ies to −17 g after year 2000, and was no longer significant for the last period (Table 2). The risk of BW < 2500 g was significantly higher in newborns of IJD women from 1980 to 1999 with an aOR varying from 1.4 (95 % confidence interval (CI) 1.2, 1.8) to 1.2 (95%CI 1.03, 1.5), but not from year 2000 (aOR 1.2 (95 % CI 0.9, 1.4)). Similarly, the risk of SGA births was higher for mothers with IJD from 1967 to 1999, and the aOR varied from 1.5 (95 % CI 1.2, 1.9) to 1.2 (95 % CI 1.1, 1.4). After year 2000 the risk of SGA was not significantly different between IJD females and references (aOR 1.1 (95%CI 0.9, 1.2)). However, the risk of prematurity was significantly higher among offspring of IJD mothers from 1980 onwards (Table 3). The risk varied from 1.5 (95 % CI 1.2, 1.8) in the 1980ies to 1.3 (95 % CI 1.1, 1.5) from year 2000.

**Table 1:** Maternal age, parity and smoking habits in births of females with IJD and reference births from the general population by year of delivery, Norway 1967-2009

Period	Groups	Number of births per period n	Proportion of births in women with IJD compared to references (%)	Maternal age at delivery (years) mean (SD)	*p*-value	Parity mean (SD)	*p*-value	Smoking at time of conception (%)	*p*-value
1967-1979	IJD women	645	0.08	28.2 (5.6)	<0.001	1.0 (1.1)	0.6	n.a.	
	References	773563		26.2 (5.3)		1.0 (1.1)		n.a.	
1980-1989	IJD women	1613	0.3	28.3 (5.1)	<0.001	0.8 (0.9)	0.2	n.a.	
	References	517621		27.3 (5.0)		0.9 (1.0)		n.a.	
1990-1999	IJD women	2508	0.4	29.7 (5.2)	<0.001	1.0 (1.1)	0.007	n.a.	
	References	581627		28.7 (5.0)		0.9 (1.0)		n.a.	
2000-2009	IJD women	2736	0.5	31.1 (5.1)	<0.001	1.0 (1.1)	<0.001	22.9	*p* < 0.0001
	References	564299		30.0 (5.1)		0.9 (1.0)		19.4	

*IJD* inflammatory joint disease, *SD* standard deviation, *n.a*. not applicable

**Table 2:** Mean birth weight in newborns of females with IJD compared with reference births from the general population by year of birth, Norway 1967-2009

Period of birth	Group	Total n	Birth weight* (gram) mean (SD)	*p*-value *	Mean difference in birth weight**(gram) mean (SD)	95 % CI	*p*-value**
1967-1979	IJD	645	3404.7 (626.1)	<0.001	−61.2 (19.7)	−99.9, −22.5	0.002
	Ref	773563	3486.9 (585.3)				
1980-1989	IJD	1613	3428.9 (634.5)	<0.001	−43.6 (12.6)	−68.3, −18.7	0.001
	Ref	517621	3512.9 (584.5)				
1990-1999	IJD	2508	3477.2 (626.5)	<0.001	−48.5 (10.3)	−68.7, −28.3	<0.001
	Ref	581627	3552.5 (600.3)				
2000-2009	IJD	2736	3500.8 (590.0)	<0.001	−16.8 (8.7)	−33.8, 0.1	0.05
	Ref	564299	3551.0 (590.9)				

*t-test **Multiple linear regression analysis with covariates for gestational age, maternal age at delivery, gender and parity

*IJD* inflammatory joint diseases, *Ref* references, *SGA* small for gestational age, *OR* odds ratio, *CI* confidence interval

**Table 3:** Newborns with birth weight < 2500 grams, SGA and preterm delivery in females with inflammatory joint diseases compared with reference births from the general population by year of birth, Norway 1967-2009

Period of birth	Group	Total *n*	Birth weight < 2500 gram					SGA					Preterm birth				
			*n*	%	OR^a^	95 % CI	*p*-value	*n*	%	OR^a^	95 % CI	*p*-value	*n*	%	OR^a^	95 % CI	*p*-value
1967-1979	IJD	645	37	5.7	1.4	1.0, 1.9	0.07	109	16.9	1.5	1.2, 1.9	<0.001	37	5.7	1.1	0.8, 1.5	0.53
	Ref	773563	32962	4.3				91 495	11.8				40 475	5.2			
1980-1989	IJD	1613	91	5.6	1.4	1.2, 1.8	0.001	201	12.4	1.2	1.04, 1.40	0.01	117	7.2	1.5	1.2, 1.8	<0.001
	Ref	517621	20250	3.9				54 770	10.6				25 354	4.9			
1990-1999	IJD	2508	119	4.7	1.2	1.03, 1.5	0.03	267	10.6	1.2	1.1, 1.4	0.001	180	7.2	1.4	1.2, 1.6	<0.001
	Ref	581627	22541	3.9				51 705	8.9				30 635	5.3			
2000-2009	IJD	2736	117	4.3	1.2	0.9, 1.4	0.12	210	7.7	1.1	0.9, 1.2	0.31	195	7.1	1.3	1.1, 1.5	0.001
	Ref	564299	20907	3.7				41 094	7.3				31 552	5.6			

*IJD* inflammatory joint diseases, *Ref* references, *SGA* small for gestational age, *OR* odds ratio, *CI* confidence interval

^a^Adjusted for maternal age at delivery and parity

## Discussion

Since 1967 births among females with IJD have significantly increased. The low number of births during the earliest period may have several explanations. In accordance with previous reports females with IJD may have been advised not to have children [7, 8]. After 1990 new treatments have evolved and knowledge has been gathered about the use of anti-inflammatory drugs before and during pregnancy. Thus more women with inflammatory active disease have been able to plan pregnancy. Biological agents like the TNF-α inhibitors, that target cytokines and immune cells, were introduced in Norway in 1999. TNF-α inhibitors are effective inhibitors of disease activity and are now recommended used until conception [12]. High inflammatory disease activity has a negative effect on sexual function [13], and better treatment options may also improve the sexual function and the possibility to achieve pregnancy. Risk of developing IJD increases with age, and the age of primiparous Norwegian women has increased since 1967 and is currently 28.5 years [14]. Thus, the higher proportion of births in females with IJD may be caused by the general increase in maternal age as well as improved documented safety of medical treatments.

We observed that the mean BW in newborns of IJD women approached that of the reference women during the study period, and the mean difference was no longer statistically significant after year 2000. The proportion of newborns with BW <2500 grams declined from 6 % during 1967 – 1979 to 4 % after year 2000 in females with IJD. Correspondingly, the SGA proportion declined from 17 % to 8 %. Interestingly, these declines coincide with the introduction of better treatment options. Low BW has been associated with high disease activity at conception and during pregnancy [1, 2]. TNF-α inhibitors may be administrated up to time of conception [15, 16] and may help more women with inflammatory active IJD to stay in or close to remission until they conceive. Better knowledge of the use of other anti-inflammatory drugs in pregnancy such as prednisone, hydroxychloroquine and sulfasalazine may also have influenced the results [16, 17]. For females with IJD Norwegian national guidelines suggest regularly surveillance in pregnancy from general practitioners, rheumatologists, obstetricians and other specialists when necessary [18]. The improved care taking and monitoring of pregnant women with IJD may have influenced our results. Ultrasound surveillance of fetal growth has become more widespread over the study period, and the use of ultrasound to detect intrauterine growth restriction may have had an impact on low BW and SGA. The proportion of preterm deliveries from 1979 onwards was 7 % in IJD women compared to around 5 % in reference women. Thus, the observed difference in preterm birth has persisted over the years despite the introduction of new effective drugs. In the earliest period (1967–1979) the number of cases was insufficient for interpretation. In a Danish population based study spanning from 1977 to 2008, offspring of females with RA had a 1.5 higher risk of preterm birth than in the general population [19]. The study did not examine secular trends. The newborns of RA women also had a slightly lower BW [19]. The observed increased risk of preterm birth was present both in females diagnosed with RA and in those with preclinical RA. The latter group had not been exposed to any anti-rheumatic medication. The study hypothesized that potential disease-induced factors influenced the observed differences in fetal growth and preterm birth. These factors are still unknown. A higher rate of preterm birth was also reported in another study of females with RA spanning from 2001 to 2009. Among 46 pregnancies 28 % delivered prior to 37 weeks [20]. The study did not find any associations between preterm birth and active disease at conception or during pregnancy. However, discontinuation of medication because of pregnancy was associated with a significantly earlier gestational age at delivery. We did not have information about drug exposure in pregnancy, and isolating the effect of disease versus treatment is difficult in our material. A few studies have indicated that use of prednisone, even in low dose may induce preterm delivery [2, 21]. Prednisone has been one of the most frequently used medications to treat inflammation in pregnant arthritic females during the last decades, but probably not in the late sixties and early seventies when the use of prednisone was sparsely documented in pregnancy [22–25]. More women with IJD were smoking at time of conception during 2000–2009, but we did not have information about smoking before year 2000. Thus, it is unknown how smoking might have influenced the results overall.

An advantage of the present study was the availability of population based data from a national registry. This limits selection bias of IJD cases and ensures representative references.

Among potential limitations we cannot exclude the possibility of misclassification bias. However, a validation study of a selection of rheumatic diagnoses in MBRN including inflammatory arthritic diagnoses, reported that 97 % of the diagnoses were correct [26]. We excluded unspecified arthritic diseases, inflammatory connective tissue diseases and vasculitis in the selection of IJD cases (Additional file 1). The birth outcomes of mothers with specific IJD diagnoses were not possible to study separately. During the period with ICD-8 coding, all IJD diseases were grouped together, and the number of cases in each diagnostic group of ICD-10 coding was not large enough. Another possible limitation is underreporting of diagnoses. MBRN has validated 169 females with rheumatic diagnoses in the period 1967–1995. Overall, 10 % of pre-pregnant diagnoses were underreported. However, underreporting was most pronounced during the earliest period (1967–1976) when it was 45 % [26]. Underreporting may certainly have led to an underestimate of births in IJD women especially during the earliest study decade. Finally, we lacked detailed information such as socioeconomic status and clinical data regarding body mass index, disease activity and medication in pregnancy. Information about smoking habits was only available after 1999.

## Conclusion

An increasing number of births among females with inflammatory joint disease have been observed between 1967 and 1999. Birth weight was lower in newborns of mothers with inflammatory joint disease than in the general population until 1999, but not later. However, an increased preterm birth rate seems to persist.

## Abbreviations

aOR, adjusted Odds ratio; BW, birthweight; CI, confidence interval; ICD, international classification of diseases; IJD, inflammatory joint disease; JIA, juvenile idiopathic arthritis; MBRN, medical birth registry of Norway; PSA, psoriatic arthritis; RA, rheumatoid arthritis; SA, spondylarthritis; SD, standard deviation; SGA, small for gestational age; TNF, tumor necrosis factor.

## Additional file

Additional file 1: Appendix with excluded codes in the patient and reference populations, according to the ICD-8 and ICD-10 systems. (DOC 24 kb)
