# Supplementary material for: Reproductive trends in females with inflammatory joint disease
Source: BMC Pregnancy Childbirth. 2016 May 31;16:123. doi: 10.1186/s12884-016-0919-7 (PMC4886403; doi:10.1186/s12884-016-0919-7)
Supplement: Additional file 1: — Appendix with excluded codes in the patient and reference populations, according to the ICD-8 and ICD-10 systems. (DOC 24 kb) [file 12884_2016_919_MOESM1_ESM.doc]

**Appendix**:

Excluded ICD codes from both patient and reference groups: ICD-8 codes 715 unspecified inflammatory arthritis, 734 (systemic lupus erythematosus, Sjøgren’s syndrome), 716 (polymyositis, dermatomyositis). ICD-10 codes systemic lupus erythematosus (M32.1, M32.8, M32.9), Sjøgren’s syndrome (M35.0), mixed connective tissue disease (M35.1), systemic sclerosis (M34.0, M34.1, M34.2, M34.8, M34.9), poly / dermatomyositis (M33.0, M33.1, M33.2, M33.9), other specified connective tissue disease (M35.8), unspecified connective tissue disease (M35.9), Still’s Disease (M06.1), unspecified arthritis (M13.0, M13.1, M13.8, M13.9), polyartheritis nodosa (M30.0), Wegener’s granulomatosis (M31.3), Takayasu’s disease (M31.4), microscopic polyangiitis (M31.7), and Behcet’s disease (M35.2). In addition also 712 (rheumatoid arthritis, juvenile rheumatoid arthritis, ankylosing spondylitis), rheumatoid arthritis (M05.0, M05.1, M05.2, M05.8, M05.9, M06.0, M06.8, M06.9), ankylosing spondylitis / spondylarthritis (M45, M46.0, M46.1, M46.8, M46.9), psoriatic arthritis (M07.0, M07.1, M07.2, M07.3 (+L40.5 for all diagnoses), juvenile idiopathic arthritis (M08.0, M08.1, M08.2, M08.3, M08.4, M08.8, M08.9), were excluded from the reference group.
